# The Environmental Protection Agency’s Use of Community Involvement to Engage Communities at Superfund Sites

**DOI:** 10.3390/ijerph16214166

**Published:** 2019-10-29

**Authors:** Larry J. Zaragoza

**Affiliations:** Environmental Protection Agency, Office of Superfund Remediation and Technology Innovation, 1200 Pennsylvania Ave NW, 5204P, Washington, DC 20460, USA; zaragoza.larry@epa.gov

**Keywords:** superfund community involvement, superfund cleanups, brownfields, revitalization, public participation, site assessment

## Abstract

The Environmental Protection Agency’s (EPA) Superfund program was established to identify, assess and clean up the nation’s worst hazardous waste sites to protect human health and the environment. Community involvement is an important part of the Superfund program for at least three reasons. First, involving communities in decision making at Superfund sites is a statutory requirement. Second, community involvement is important so that clean up decisions will support reuse in the surrounding community. Third, because even after cleanup many sites have residual contamination that warrants administrative and legal controls to protect health and the environment, community members should understand these controls to both help protect community members and any limitations on site reuse. Community feedback informs both proposed actions and local reuse decisions. While the EPA recognizes that the agency performs many activities that are helpful to support community involvement, there are areas in need of improvement and further research would be helpful for communities in the future.

## 1. Introduction

The Superfund program serves as an example of how the federal government involves communities as it works with a variety of stakeholders in the cleanup process. The program defines community involvement as the process of engaging in dialogue and collaboration with community members. The goal of Superfund community involvement is to advocate and strengthen early and meaningful community participation during Superfund cleanups. While some of the work associated with cleanup is undertaken by states and tribes through cooperative agreements, the federal Superfund program has not been delegated to states.

The Superfund program has four overarching goals, which are:

- protect human health and the environment by cleaning up contaminated sites;
- make responsible parties pay for cleanup work;
- involve communities in the Superfund process; and
- return Superfund sites to productive use.

In the early days of the Environmental Protection Agency (EPA), it had a challenging situation in gaining public trust because of the health-related impacts of contaminants reported from some communities. Perhaps the most well-known of these hazardous waste sites is the Love Canal site. The site was created from the disposal of more than 21,000 tons of hazardous chemicals in the abandoned Love Canal Landfill from 1942 to 1953 [1]. Then the landfill was covered with soil and the property was used for the construction of a school and developed into a residential area. Complaints about odors and residues were first reported during the 1960s and these complaints grew with time. The federal authority to address these problems was provided through the passage of the Comprehensive Environmental Response, Compensation, and Liability Act of 1980 (CERCLA or “Superfund”) and adoption of the National Oil and Hazardous Substances Pollution Contingency Plan [2] and program guidance.

Involving communities in the cleanup process can bring diverse stakeholders to the dialogue on site cleanup. The early community involvement program was criticized as deficient [3]. Some of the concerns raised include: failure to address the impacts on environmental justice communities; failing to provide adequate information on cleanup activities so that communities could engage; taking longer to clean up environmental justice communities; selectively responding to communities that can afford legal support to obtain EPA’s attention and action; failure to consider more permanent remedies (i.e., remedies that remove or destroy contamination so that there will be no residual waste that poses a threat that would require post construction management); and administering the Technical Assistance Grant (TAG) program so that it is too burdensome for grant recipients. The scope of these concerns extends to areas beyond community involvement (e.g., the use of remedies that are not permanent). Recognizing that improvements could be made to better engage communities, multiple changes were undertaken in the program, which are described in this paper. In a review of Superfund community involvement work, Charnley and Engelbert [4] stated that EPA’s view is that “EPA believes that cleanup efforts will be most successful if people are well informed about them, have early and meaningful opportunities to provide input about what is being done, and are able to help shape the decisions being made.” This belief remains true today and has continued to guide community involvement work. 

### 1.1. Superfund Remedial Process

**Figure 1:**
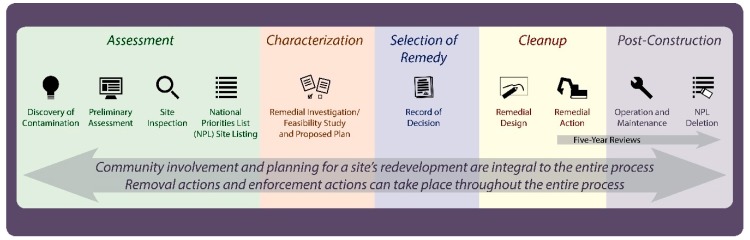
The Superfund Remedial Process.

In order to provide context for the community involvement activities that are undertaken, it is helpful to review the Superfund process. Figure 1 [5] shows the Superfund process, which has been modified from a figure routinely used for outreach and communication activities by EPA. Sites typically enter the Superfund process when they are referred to EPA by state agencies.

Removal actions and enforcement activities can also take place throughout the lifecycle of a Superfund site. When an imminent threat to the public must be addressed, time critical or emergency response removals can be undertaken. Time-critical and emergency removal responses address urgent threats, therefore these removal actions can be ordered and implemented over a very short timeframe. Non-time critical removals, which have additional public participation requirements over time critical or emergency removal actions, are also employed to address cleanup needs. Enforcement actions include efforts to get responsible parties to either take on assessment, cleanup and any post construction activities or to pay the costs of these activities.

Discovery is followed by two steps used to screen sites to determine if further action is needed. First a preliminary assessment is performed, which typically involves conducting a review of the site’s history and examining the condition of the site. Second, if it appears that the site warrants further investigation, a site inspection is conducted, which will involve sampling of potentially contaminated media and an evaluation of people and environmental resources that might be impacted. This information is used to score a site using the Hazard Ranking System, a tool used to evaluate whether a site is placed on the National Priorities List (NPL). Placement on the NPL is important because sites on the NPL are eligible for remedial funding and more community involvement support than sites not on the NPL. NPL sites are considered to be among the nation’s worst hazardous waste sites.

Site characterization, remedy selection and cleanup are the next steps in the Superfund process. Sites are characterized in a remedial investigation. Cleanup alternatives are evaluated in a feasibility study. Following the completion of these steps, a proposed cleanup plan is developed, and the selection of a cleanup remedy is documented in a record of decision. When complete, the cleanup should support the planned land use. However, if waste is left in place, post construction activities will be needed to manage residual waste, which could pose a risk to people or the environment.

### 1.2. Other Land Cleanup Programs and Community Involvement

The EPA land cleanup programs, including the Brownfields, the Resource Conservation and Recovery Act, and the Underground Storage Tank programs, all share an interest in promoting site reuse and recognize the necessity of information and working with local communities to help design cleanups that will be effective for site reuse. CERCLA amendments established the Brownfield program to support and provide technical assistance to community driven assessment and cleanup at the numerous, lower risk vacant, abandoned and underused contaminated properties. The Brownfields program targets addressing sites in communities with abandoned manufacturing, industrial and commercial properties that are not as contaminated as Superfund sites. They employ similar community involvement approaches and techniques to address property-specific environmental issues and contribute to community solutions for the array of environmental and public health issues and economic challenges, such as strengthening workforce skills to meet local job market environmental needs [6].

### 1.3. Objective

This paper provides a perspective on the development of today’s community involvement program and describes how EPA has sought to improve the Superfund community involvement program.

## 2. Materials and Methods

This section describes improvement to the Superfund’s community involvement program that have resulted from input from communities, local officials, states and tribes. Many of the features of today’s Superfund community involvement program did not exist in the early years of the Superfund program and there has been an effort to continually improve the tools to support communities. The Superfund community involvement program has worked with community relations practitioners in both the commercial and academic communities to improve our community involvement program to better serve the needs of impacted communities.

The information that has been used to inform adjustments to the community involvement program has largely resulted from several efforts. First, EPA regional staff choose from a broad selection of approved questions to evaluate how community members at their site feel about the effectiveness of the community involvement program. Second, EPA staff and managers meet with community members, elected officials and others and receive feedback that helps to identify both strengths and weaknesses. Third, the EPA is also informed by the work of researchers that perform community involvement work at the site level or perhaps a national level.

### 2.1. New and Improved Tools to Support Communities

The cleanup of Superfund sites is a complex process that uses a lot of technical information and legal considerations. Community members often find issues like the extent of contamination, the toxicity of chemicals and the possible methods to clean up a site to be challenging. Also, it can be hard to see why it might not be feasible to clean some sites to unlimited use and unrestricted exposure (i.e., a cleanup that leaves no residual waste at a level that does not pose an unacceptable risk). Table 1 highlights key community involvement tools, which have been summarized from the EPA’s Superfund community involvement website. These tools help communities understand the cleanup process and find their voice to meaningfully participate in site cleanup discussions.

As outlined in the Community Involvement Handbook [8], Community Involvement Plans (CIPs) form the basis for the identification of community involvement needs and the plans to address those needs. The CIP guidance calls for preparation of a CIP and public notification of work to be undertaken before the first round of sampling to support the remedial investigation is performed at a site. CIPs are updated as new information becomes available and as a site progresses through the assessment, characterization, cleanup and post construction activities. Technical Assistance Needs Assessments (TANAs) help to scope appropriate CIP components and site activities to better address community needs.

While TAGs have been a part of the community involvement program before 1990, the other tools listed have been developed to address community involvement needs. EPA also offers Technical Assistance Services for Communities (TASC) contractor support to review and summarize information for communities, which eliminates the administrative burdens of managing a grant for a community group. Mediation services are also provided to assist communities, which can be helpful when there are divergent views regarding site cleanup issues. Mediation services, especially when coupled with expert advice, has been helpful to many communities to come to agreement on future land uses and to provide the community greater comfort that the cleanup will be protective of their health.

### 2.2. Public Notification

Recognizing that the CERCLA statute was last amended in 1986 and that there has been a shift from newspapers to electronic media to obtain news, EPA amended the National Oil and Hazardous Substances Pollution Contingency Plan to broaden the mechanisms that could be used to inform communities of CERCLA actions from newspapers to fact sheets, flyers, letters/postcards, social media, telephone calls, posting information on websites, asking community members to post information on neighborhood listservs, emailing notices and issuing regional press releases to media outlets. The statute requires publication of the events listed below in newspapers. Table 2 below summarizes public notice requirements under Superfund, which may take the form of providing information to communities, publication in the Federal Register, or publication in a newspaper.

### 2.3. Environmental Justice

Superfund community involvement identifies and supports all communities, including environmental justice communities. EPA defines environmental justice as the fair treatment and meaningful involvement of all people regardless of race, color, national origin or income with respect to the development, implementation and enforcement of environmental laws, regulations and policies [10]. One of the first steps in evaluating sites for potential environmental justice concerns typically involves the use of EPA’s EJSCREEN (an environmental justice screening and mapping tool that provides a nationally consistent approach to characterizing areas that may warrant further consideration, analysis or outreach as environmental justice communities) to provide demographic information surrounding a site [11]. EJSCREEN is a useful tool to help assess community demographics so that outreach activities can better support the community for meaningful participation in the cleanup process. While EPA offers additional support through translation services or technical support or additional outreach to meaningfully participate in site cleanup activities for all communities, environmental justice communities are especially likely to benefit from this support.

### 2.4. Training

The Community Involvement University helps to prepare those individuals who work with communities to better meet community needs. Training for EPA staff as well as state, tribal and local partners is also an important part of today’s community involvement program [12]. The Superfund program offers a wide range of training from basic community involvement skills to advanced facilitation skills for EPA staff as well as state and local partners. This training also helps practitioners to develop communication skills to effectively work with communities at contaminated sites.

### 2.5. Information Technology

NPL sites have extensive records that are shared with the public through websites and administrative record repositories. The record repositories are typically located near sites and at regional offices. Superfund site information is summarized on the Superfund site profile of each NPL. NPL site information available on the Superfund site profile pages include site status, documents and background information. Superfund site profile pages are readily accessible through site search results [13]. Many past site activities and other cross-media environmental information for EPA’s land cleanup programs can be found on EPA environmental data platforms, such as EPA’s Envirofacts and Cleanups in My Community.

### 2.6. Site Reuse

While site reuse and community involvement are separate activities, they both involve working with many of the same stakeholders and EPA staff. Consideration of site reuse is triggered by the need to plan a cleanup to support future land use. Time has shown that by engaging communities in reuse planning, the likelihood that reuse plans will be implemented are increased.

## 3. Results

While various community groups and even national organizations interested in community involvement have observed that the EPA has made significant progress to support communities so that they can engage in meaningful dialogue affecting the future of impacted communities, community involvement challenges remain. Today’s community involvement tools are more effective in the assistance of addressing community concerns. However, even with better tools, it can still take a long time to resolve community involvement concerns at sites.

Recognizing that there were many barriers to community participation, EPA’s Superfund community involvement program has developed tools to identify and remove barriers to participate in the cleanup process. The program operates with the expectation of working with communities to improve their understanding of the Superfund process and site risks will increase community participation. Targeting community involvement services to the needs of communities has helped to provide needed services. Moreover, efforts to provide services so that communities do not need to obtain a TAG to obtain technical services relieves administrative burdens. At the same time, communities may obtain a TAG if they are willing to take the necessary steps, which are largely determined by federal and EPA-wide procurement rules.

## 4. Discussion

As noted in the introduction, the 4th goal of the Superfund Program is to return sites to productive use. Timing is a consideration in cleanups as neither the EPA nor communities want the cleanup process to be unnecessarily protracted. Community involvement can be helpful in building support for cleanup plans to move forward in a timely manner. However, while community acceptance is important, the site owner will ultimately control site reuse.

**Figure 2:**
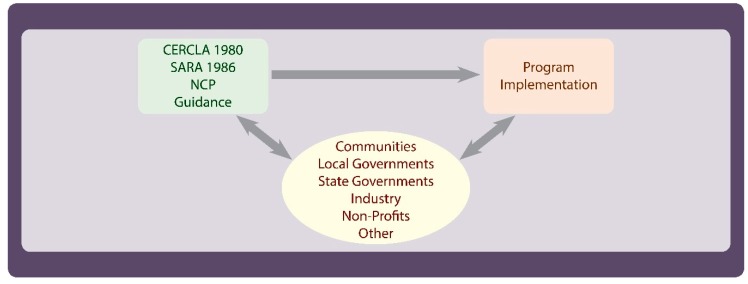
Program Policy, Implementation and Public Input.

Figure 2 highlights the role of public input, which may include communities, states, tribes, industry (including specific parties that were responsible for site contamination), special interest groups and possibly others. As noted earlier, the CERCLA statute was adopted in response to public concern over hazardous waste sites and their impact on the health of communities. To implement the CERCLA statute, regulations were adopted which provide additional specificity for program implementation. Further specificity was provided in program guidance, which provides more details to assist with program implementation. Moreover, states, local governments and tribes are partners in the implementation of cleanup programs and their views are sought in the development of environmental cleanup programs.

While there are many groups that comprise the public, communities have historically been one of the most influential in the Superfund process. They were instrumental in influencing Congress and the President to adopt the CERCLA statute. Throughout the history of the program, communities have captured headlines, and provided key information used to make decisions on cleanup and future land uses. When communities are not satisfied, they have and continue to raise concerns to the agency, the press and Congress. While communities have a profound influence on EPA, they also significantly influence their local, state and tribal governments.

### 4.1. Engaging Communities

While there are still community involvement concerns, the community involvement program helps communities to better engage in the cleanup process today. There are reasons why a community may or may not actively follow site cleanup activities and it can be difficult to predict how a community will or will not participate in the cleanup process. In evaluating the role of trust in the restoration process, Metcaff et al. [14] evaluated reactions from a wide range of stakeholders and concluded that trust is a key factor that appears to support effective collaboration. However, as noted by Probst [15], Superfund sites vary greatly in their characteristics and these differences make it difficult to compare sites without controlling for variables that influence the cleanup process.

Communities are more likely to actively participate when they understand the issues and believe they can influence an outcome. This belief is supported, at least to some extent, by research. Slovic has examined the relationship between familiarity and concern for known risks (e.g., risk of walking across a road against a red light). His findings report that there tends to be a lower concern for activities with a known risk, such as walking across the street against a stoplight yet risk from hazardous chemicals or radiation may be viewed with greater concern even when the risk is lower. He concluded that participation in the evaluation and management of risks will lead to more satisfying ways to manage risk [16]. This conclusion is consistent with the findings of Charnley and Engelbert who determined that communities who understand the contamination and cleanup issues are more likely to be supportive of the cleanup efforts.

Even though community members know that cleanup decisions will affect their health, safety and property values, they may not choose to participate in the process. There may be a number of reasons for this behavior such as meeting times conflict with work, they believe others will effectively represent their interests, or they do not think their input will be used. The process of engagement may seem to be difficult to community members. Thus, they may decide not to participate [17].

### 4.2. Cleanup Partners

CERCLA designates state and tribal governments as cleanup partners with the EPA in the identification, assessment, cleanup and post cleanup monitoring of Superfund sites. Coordination with these partners is important to improve messaging and to identify possible future issues. In general, there is a widely held expectation that government agencies should coordinate amongst themselves as communities should not be expected to distinguish messages from different agencies. Reconciling differences in information among different partners helps to provide a more coherent message to communities.

Superfund’s cleanup partners hold different responsibilities that complement the EPA’s work. Given the importance of local governments in reuse planning and the adoption and implementation of institutional and engineering controls to address residual waste, local, state and tribal governments are important partners for cleanups. Also, state and tribal legislatures adopted laws to identify and address abandoned contaminated sites and establish complementary laws and programs for response and cleanup.Coordination with Agency for Toxic Substances and Disease Registry (ATSDR) has helped so that (1) the EPA and ATSDR both have an appreciation of their respective activities and (2) the presentation of information to the public is less confusing because any differences between the EPA and ATSDR are more clearly communicated. ATSDR is required to address public health issues at Superfund site and they provide outreach to communities to explain the results of their investigations. ATSDR works with the EPA and often funds state and local health agencies to support health work near Superfund sites.

As part of the Superfund amendments of 1986, Congress created the Superfund Research Program with the National Institutes of Environmental Health Sciences (NIEHS). This is a robust research program that includes basic and applied science as well as community outreach and education components. This research helps to inform Superfund work and has established communications with researchers and NIEHS program staff to facilitate information exchange [18].

To promote coordination the EPA has worked with NIEHS and their grant recipients to develop a model memorandum of agreement for work with NIEHS grantees. This model agreement outlines the Partners in Technical Assistance Program (PTAP). The PTAP program provides a process for researchers to work with site managers at Superfund sites. The PTAP program simplifies issues such as site access and coordination with site managers, which might otherwise pose some logistical challenges to universities and other academic institutions that wish to conduct studies at Superfund sites. The PTAP program provides a promising vehicle to bring researchers together with EPA staff to support communities. The exchange of information between EPA staff and grantees that takes place during PTAP projects also helps grantees to better focus their research to areas that have a bigger impact on advancing the underlying science and community involvement practices supporting site cleanups.

### 4.3. Benefits of Site Reuse

Promoting site reuse not only serves as a catalyst to bring different community stakeholders together, site reuse spurs economic benefits. According to the 2018 Superfund annual accomplishment report [19], Superfund site reuse is estimated to provide annual employment benefits of $13 billion, over 195,000 jobs and supports 8,600 businesses. However, the reuse of sites has another critical benefit. It is helpful to have active parties onsite when there is residual waste left in place who can quickly notify appropriate parties should institutional controls or engineering controls be at risk, which also benefits communities.

### 4.4. Translation of Research

Translating the results of research into outcomes that help communities engage in programs like Superfund or aid in informing cleanup decisions through research on toxicity or treatment technologies has played a role in changing the Superfund program. Research has resulted in changes to the known toxicity of contaminants that determine cleanup levels and the use of new cleanup technologies. The Superfund program engages with researchers, including the NIEHS Superfund Research Program, that study community engagement, contaminant toxicity and treatment technologies. This coordination promotes a shared understanding of relevant research that is ongoing and helps researchers to better understand where research is likely to be most helpful in advancing environmental policies for the Superfund program.

Recognizing the need for the exchange of scientific information for scientists, the advancement of technologies, communications and policy development, NIEHS has developed a Research Translation Framework [20]. This framework refines earlier work to track new ideas. Translating research from basic research to applied research or changes in policy is and has been challenging and this framework may help to inform research design, policy development and reduce impacts.

## 5. Conclusions

Today’s Superfund community involvement program provides robust support for communities to support them in finding their voice to participate in site related decisions. In order for community involvement to be most effective in influencing site cleanup and reuse decisions, it is important that the various legal and administrative constraints associated with a site be understood and that input be provided before these decisions are made. To facilitate community involvement participation, the program has reduced the administrative burden to communities to obtain support and a wide range of services from technical experts to third party mediation and other facilitation services are available. In order to prepare community involvement, state, tribal and local staff to work with communities, a rich training program is available and classes are offered in different parts of the country to make the training more accessible. While there is clear progress in the community involvement program, there still are opportunities for improvements to be made in the future.

Partnership with state and local governments and with other federal agencies like ATSDR has also been beneficial in promoting effective community involvement support. Such coordination helps to make the presentation of information clearer and reduces confusion that would otherwise be evident when information is not coordinated.

Superfund reuse planning brings various stakeholders together and helps to promote efforts to select remedies that will be protective and integrate community input into the decision-making process. By integrating community input into the site decision-making process, it is more likely that communities will have a vision for the future use of the contaminated land and that the cleanup will be protective for desired uses. Moreover, reuse planning has often been helpful for communities to have better services and community revitalization than they would not have experienced without reuse planning.

Community involvement is similar to other areas of research on hazardous waste sites where the sharing of information from researchers and dialogue to fully appreciate the research helps to inform policy development. Moreover, the exchange of information with policy makers helps researchers design research projects that will be more likely to influence and improve future policies.

## Figures and Tables

**Table 1:** Methods that the Environmental Protection Agency (EPA) employs to promote community participation [7].

Tool	Description
Technical Assistance Needs Assessment (TANA)	A TANA helps the EPA determine what technical assistance resources and information the Agency can provide to meet community needs.
Technical Assistance Services for Communities (TASC)	An EPA contractor provides scientists, engineers and other professionals to review and explain information to communities. TASC services are determined on a project-specific basis and provided at no cost to communities.
Partners in Technical Assistance Program (PTAP)	This program provides a mechanism for colleges and universities to voluntarily provide technical support to communities.
Technical Assistance Grant (TAG)	TAGs are awarded to non-profit community groups to contract with contractors to provide independent technical support to communities.
Technical Assistance Plan (TAP)	A TAP is funded by potentially responsible parties through a negotiated settlement agreement. The funding enables a community group to retain the services of an independent advisor and provides resources for the community group and other to learn about decisions and participate in the cleanup process.

**Table 2:** Summary of required public notice requirements for Superfund sites [9].

Type of Site Action	Event	Newspaper Publication Required
Time Critical Removal Action	The administrative record file becomes available for a time-critical removal action (40 CFR 300.415(n)(2)(i)).	
Non-Time Critical Removal Action	The analysis (i.e., Engineering Evaluation/Cost Analysis (EE/ CA)) to support a non-time critical removal action is issued (40 CFR 300.415(n)(4)(ii)).	
Non-Time Critical Removal Action	The administrative record file becomes available for a non-time critical removal action (40 CFR 300.820(a)(1)).	
Any Removal Action	The administrative record becomes available for any other removal actions (40 CFR 300.820(b) (1)).	
NPL Site Status	A site is proposed to be deleted from the NPL (40 CFR 300.425(e)(4)(ii)).	
NPL Site Record	The administrative record file for the selection of a remedial action becomes available at the start of the remedial investigation (40 CFR 300.815(a)).	
NPL Site Proposed Plan	The Proposed Plan for a cleanup becomes available (CERCLA §§117(a)(1) and 117(d)).	•
NPL Site Cleanup Plan Decision	The cleanup has been selected and the ROD is signed (CERCLA §117(b) and 117(d)).	•
NPL Site Enforcement Actions	A remedial action, CERCLA 106 enforcement action, or CERCLA 106/122 settlement is taken that differs significantly from the remedial action that had previously been selected and documented in the ROD or in any ROD amendments (CERCLA §117(c) and 117(d)).	•
NPL Site Assistance	40 CFR 35.4110 calls for public notification in a “major newspaper of general circulation” after EPA receives a letter of intent to apply for a Technical Assistance Grant (TAG).	•
